# Revision of the genus *Emphylica* Turner, 1913 based on morphology and molecular data (Lepidoptera, Crambidae, Pyraustinae)

**DOI:** 10.3897/zookeys.836.32796

**Published:** 2019-04-08

**Authors:** Kai Chen, Qingming Liu, Jianhua Jin, Dandan Zhang

**Affiliations:** 1 State Key Laboratory of Biocontrol/The Museum of Biology, School of Life Sciences, Sun Yat-sen University Guangzhou China; 2 Sun Yat-sen University, Guangzhou, Guangdong 510275, China Sun Yat-sen University Guangzhou China

**Keywords:** *
Achyra
*, China, conical frons, *
Loxostege
*, molecular phylogeny, new combinations, new species, *
Sitochroa
*

## Abstract

Moths of the genus *Emphylica* Turner, 1913 resemble species of *Achyra* Guenée, 1849, *Loxostege* Hübner, 1825 and *Sitochroa* Hübner, 1825 in having a conical frons. In order to examine the monophyly of *Emphylica*, and its relationship to other genera with a conical frons, a molecular phylogenetic framework is reconstructed based on sequence data of COI, 16S rRNA, 28S rRNA, EF-1α and Wg gene regions. The results robustly support the monophyly of *Emphylica*. *Achyra* + (*Loxostege* + *Sitochroa*) is in a sister position to *Emphylica*. A new species, *E.crassihamata***sp. n.**, is described from Southern China and two new combinations, *E.diaphana* (Caradja & Meyrick, 1934), **comb. n.** and *E.cruoralis* (Warren, 1895), **comb. n.**, are proposed. An identification key based on males is provided for all *Emphylica* species. The adult habitus and genitalia of all species are figured.

## Introduction

*Emphylica* was established by Turner (1913) and remained monotypic until now, with the type species E.xanthocrossa Turner, 1913 endemic in the north of Australia. In appearance, *E.xanthocrossa* can be best distinguished from other pyraustine species by the small wingspan (less than 15 mm), the reddish brown forewing with a yellow costal spot and the characteristic conical frons.

The frons of pyraustine species is usually flat or round, seldom projecting conically. Based on current knowledge, only the Australian genus *Emphylica* Turner, the genus *Achyra* Guenée with a worldwide distribution, the mainly Holarctic genera *Loxostege* Hübner and *Sitochroa* Hübner, as well as the New World genera *Hahncappsia* Munroe, *Neohelvibotys* Munroe and *Helvibotys* Munroe have a conical frons. These genera (except *Emphylica*) were considered closely related to each other based on external characters (Munroe 1976a).

While examining pyraustine collections from southern China and Southeast Asia, *Loxostegediaphana* Caradja & Meyrick, 1934, *Pyraustacruoralis* (Warren, 1895) and an undescribed species, all resembling *Emphylicaxanthocrossa* in the conical frons and the relatively small wingspan (less than 20 mm), attracted our attention. Further studies based on the genitalic characters suggested that the two described species definitely did not belong to their current genera and these three species were closely related to *Emphylica*. In order to evaluate the generic placement of these species, the phylogenetic relationships of *Emphylica* and potentially related genera, *Achyra*, *Loxostege*, and *Sitochroa*, were studied based on genetic data. The taxonomic composition and morphology of *Emphylica* are redefined.

## Material and methods

### Molecular phylogenetic analysis

In total eleven species of five genera were included for molecular phylogenetic analysis (Table 1). *Pseudebuleafentoni* Butler, 1881 was chosen as the outgroup because it was considered as a basal lineage of the Pyraustinae (Zhang 2003). The New World genera *Hahncappsia* Munroe, *Neohelvibotys* Munroe and *Helvibotys* were not included in the current study because no specimens could be accessed.

**Table 1.** Species sampled for the molecular phylogenetic analysis.

**Taxon**	**Voucher**	**Locality**	**GenBank accession number**					**References**
			COI	16S	28S	EF1-α	Wg	
* Pseudebulea fentoni *	SYSULEP0074	Hunan	MG739570	MG739582	MG739605	MG739594	MK506279	Chen et al. 2018; present study
* Emphylica crassihamata *	SYSULEP0190	Guangdong	MK506732	MK506755	MK506767	MK506744	MK506728	present study
* Emphylica crassihamata *	SYSULEP0191	Hunan	MK506733	MK506756	MK506768	MK506745	MK506727	present study
* Loxostege deliblatica *	SYSULEP0200	Xinjiang	MK506734	MK506757	MK506769	MK506746	MK506726	present study
* Loxostege sticticalis *	SYSULEP0227	Xinjiang	MK506735	MK506758	MK506770	MK506747	MK506725	present study
* Achyra massalis *	SYSULEP0242	Shanxi	MK506736	MK506759	MK506771	N/A	MK506724	present study
* Sitochroa verticalis *	SYSULEP0257	Xinjiang	MK506737	MK506760	MK506772	MK506748	MK506723	present study
* Sitochroa palealis *	SYSULEP0258	Xinjiang	MK506738	MK506761	MK506773	MK506749	MK506722	present study
* Emphylica diaphana *	SYSULEP0263	Fujian	MK506739	MK506762	MK506774	MK506750	MK506719	present study
* Emphylica diaphana *	SYSULEP0264	Guangdong	MK506740	MK506763	MK506775	MK506751	MK506720	present study
* Emphylica xanthocrossa *	SYSULEP0307	Western Australia	MK506741	MK506764	MK506776	MK506752	MK506730	present study
* Emphylica cruoralis *	SYSULEP0377	Tibet	MK506742	MK506765	MK506777	MK506753	MK506721	present study
* Sitochroa umbrosalis *	SYSULEPT014	Fujian	MK506743	MK506766	MK506778	MK506754	MK506731	present study

Total DNA was extracted from two legs and sometimes in addition from the abdomen of the dry specimens using the TIANGEN DNA extraction kit following the manufacturer’s instructions. The nucleotide sequences of two mitochondrial genes, cytochrome c oxidase subunit I (COI) and 16S ribosomal RNA (16S rRNA), and three nuclear genes, 28S ribosomal RNA (28S rRNA), Elongation factor-1 alpha (EF-1α) and Wingless (Wg) were selected for study. Primers used in this study are as follow: LCO/Nancy for COI and LepWg1/LepWg2 for Wg (Wahlberg and Wheat 2008), LR-J-12888/ LR-N-13398 for 16S rRNA (Simon et al. 2006), Oscar-6143/Bosie-6144 for EF-1α (Hundsdöerfer et al. 2009) and 28S-f1/28S-r1 for 28S rRNA (Lee and Brown 2008). PCR cycle conditions were set to an initial denaturation of 5 min at 95 °C, 35 cycles of 30 seconds at 94 °C, 30 seconds at 48 °C (COI, Wg and 16S rRNA) or 52 °C (EF-1α, 28S rRNA) and 1 min at 72 °C for amplification, and a final extension at 72 °C for 10 min. PCR products were confirmed with 1.5% agarose gel electrophoresis in TAE buffer, then were direct-sequenced at Majorbio Bio-pharm Technology Co., Ltd (Guangzhou), utilizing the same primers used for PCR amplification.

The sequences were aligned using Clustal W (Thompson et al. 1994) in MEGA 6 (Tamura et al. 2013) with default settings. The aligned matrix was corrected by eye. Gaps were treated as missing data. Phylogenetic analyses were inferred using Bayesian inference (BI) method in MrBayes 3.2.6 (Ronquist et al. 2012) and maximum likelihood (ML) in RAxML 8.2.10 (Stamatakis 2014). BI analysis was run with independent parameters for the COI gene partition under the GTR + I model, the 16S rRNA and 28S rRNA gene partitions under the GTR + G model, the EF-1α gene partition under the K80 + I model, and the Wg gene partition under the HKY + G model as suggested by jModelTest 0.1.1 (Posada 2008). Two independent runs, each with four Markov Chain Monte Carlo (MCMC) simulations, were performed for 10 million generations sampled every 1000th generation. The first 25% trees were discarded as burn-in, and posterior probabilities (PP) were determined from remaining trees. ML analysis was executed under the GTR + G model for all gene partitions and with 1000 iterations for the bootstrap test. The pairwise Kimura 2-Parameter (K2P) distances between species were calculated from the COI gene using MEGA 6 (Tamura et al. 2013).

**Morphological analysis.** The specimens studied, including the types of the newly described species, are deposited in the Museum of Biology, Sun Yat-sen University, Guangzhou (**SYSBM**), except for those held at the following institutions: the Insect Collection of the College of Life Sciences, Nankai University (**NKU**), the Australian National Insect Collection (**ANIC**), and the Natural History Museum, London, United Kingdom (**NHMUK**). Slides of dissected genitalia were prepared according to the protocols of Robinson (1976) and Li and Zheng (1996). Terminology of genitalia follows Maes (1995), except for the terms “phallus” and “colliculum” for which we follow Kristensen (2003). Images of the specimens were taken using a Canon EOS 1DX camera provided with a Canon 100 mm macro lens; images of specimen of *Emphylicacruoralis* were taken using a Canon EOS 5DS R camera provided with a Canon 65 mm macro lens. The genitalia pictures were taken using a Zeiss Axio Scope.A1 in combination with a Zeiss AxioCam camera and the Axio Vision SE64 program on a Windows PC; genitalia pictures of *Emphylicacruoralis* were taken using a Zeiss Axioskop in combination with a Canon EOS 700D camera and Helicon Remote. Source images were then aligned and stacked with Helicon Focus to obtain a composite image.

## Results

### Phylogenetic relationships

**Figure 1.**
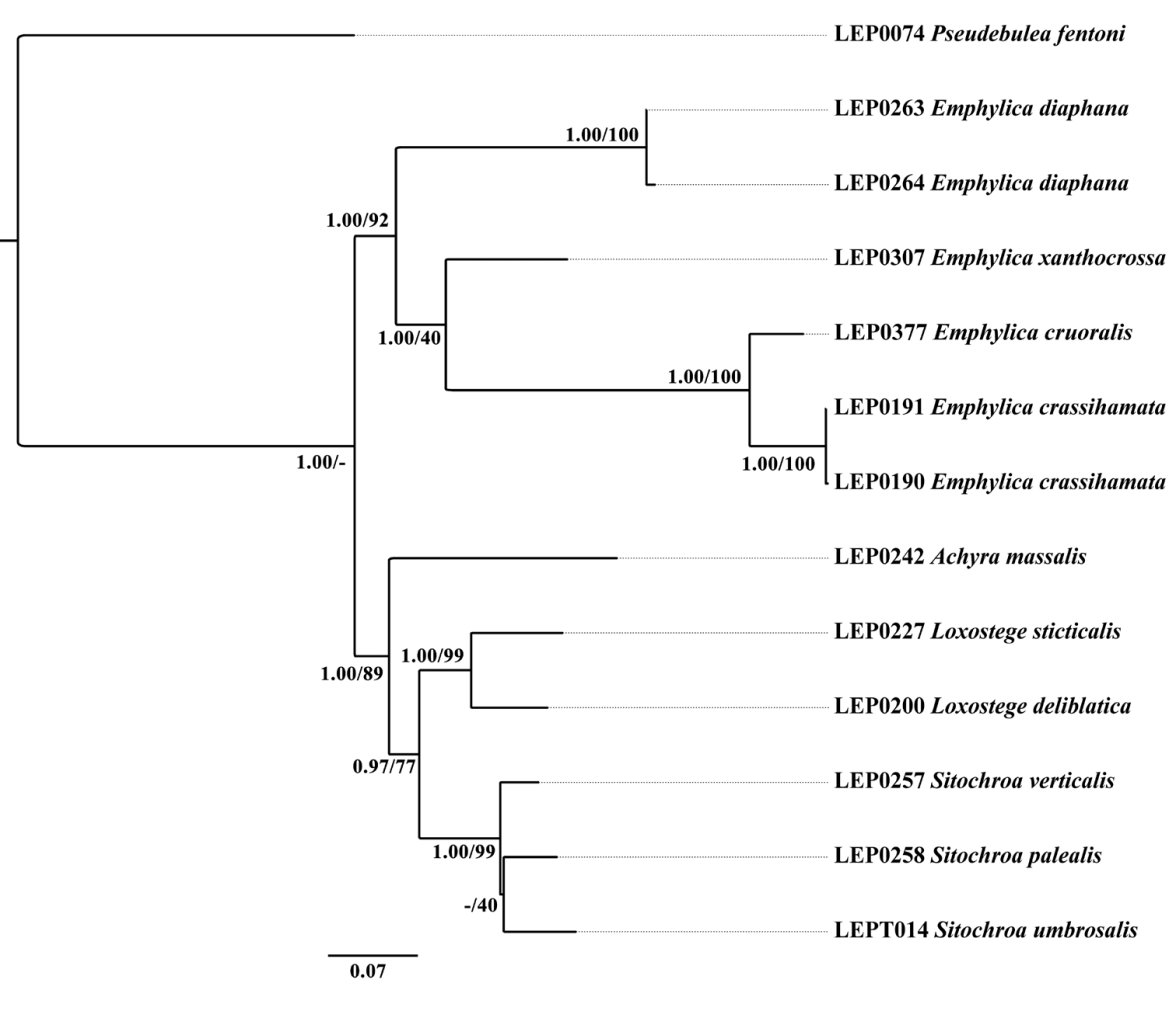
Phylogenetic hypothesis inferred from Maximum likelihood (ML) analysis. Numbers on branches indicate Bayesian posterior probabilities and ML bootstrap values, respectively.

The concatenated dataset of five genes consisted of 2873 nucleotide positions (658 for COI, 466 for 16S, 612 for 28S rRNA, 753 for EF-1α and 384 for Wg). Both BI and ML analyses of the concatenated dataset inferred congruent topologies with only subtle differences in posterior probability and bootstrap values probability (Fig. 1). The monophyly of *Emphylica* is robustly supported (PP = 1.00, BS = 92). The clade *Achyra* + (*Loxostege* + *Sitochroa*) is in a sister position to *Emphylica* with robust support (PP = 1.00).

The results of the current phylogenetic analyses support that the undescribed species (here named as *E.crassihamata* sp. n.) should be placed in *Emphylica*, and that *E.diaphana* comb. n. should be transferred from *Loxostege* to *Emphylica* and *E.cruoralis* comb. n. should be transferred from *Pyrausta* to *Emphylica*. *Emphylicaxanthocrossa* and *E.crassihamata* + *E.cruoralis* form a sister group, although with low support in the ML analysis (BS = 40).

Pairwise distances of the barcoding region (COI) are given in Table 2. The genetic distances between *Emphylica* and other genera range from 8.3% (*Loxostege*) to 14.6% (*Pseudebulea*). Interspecific genetic distances within *Emphylica* range from 4.0% (*E.crassihamata* to *E.cruoralis*) to 8.3% (*E.crassihamata* to *E.diaphana*), while intraspecific genetic distances in *Emphylica* range from 0% (*E.crassihamata*) to 0.8% (*E.diaphana*).

**Table 2.** Pairwise distances of the COI barcode region based on Kimura-2-parameter model (intraspecific distances are highlighted in bold).

		**1**	**2**	**3**	**4**	**5**	**6**	**7**	**8**	**9**	**10**	**11**	**12**
1	LEP0190 *Emphylicacrassihamata*												
2	LEP0191 *Emphylicacrassihamata*	**0.000**											
3	LEP0263 *Emphylicadiaphana*	0.083	0.083										
4	LEP0264 *Emphylicadiaphana*	0.079	0.079	**0.008**									
5	LEP0307 *Emphylicaxanthocrossa*	0.066	0.066	0.081	0.075								
6	LEP0377 *Emphylicacruoralis*	0.040	0.040	0.077	0.074	0.063							
7	LEP0200 *Loxostegedeliblatica*	0.108	0.108	0.112	0.112	0.086	0.096						
8	LEP0227 *Loxostegesticticalis*	0.102	0.102	0.087	0.085	0.083	0.090	0.076					
9	LEP0242 *Achyramassalis*	0.100	0.100	0.092	0.096	0.098	0.089	0.090	0.083				
10	LEP0257 *Sitochroaverticalis*	0.123	0.123	0.112	0.108	0.104	0.116	0.090	0.087	0.116			
11	LEP0258 *Sitochroapalealis*	0.092	0.092	0.106	0.102	0.085	0.094	0.073	0.086	0.090	0.058		
12	LEPT014 *Sitochroaumbrosalis*	0.123	0.123	0.129	0.122	0.118	0.120	0.089	0.102	0.112	0.060	0.056	
13	LEP0074 *Pseudebuleafentoni*	0.133	0.133	0.144	0.141	0.135	0.146	0.147	0.144	0.148	0.155	0.137	0.159

## Taxonomy

### Emphylica

Taxon classificationAnimaliaLepidopteraCrambidae

Turner, 1913

Emphylica Turner, 1913: 159. Type species: Emphylicaxanthocrossa Turner, 1913, by monotypy.

#### Diagnosis.

**Figures 2–10.**
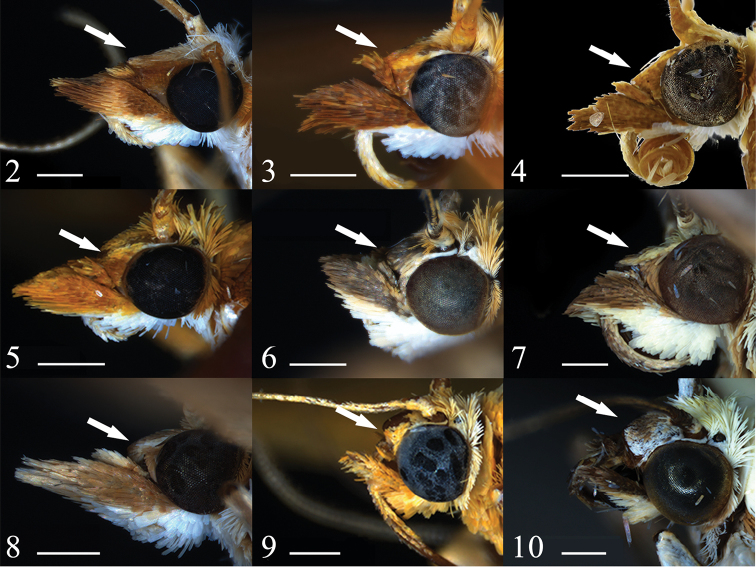
Head profiles of species of *Emphylica*, *Loxostege*, *Achyra and Sitochroa***2***Emphylicadiaphana***3***E.crassihamata***4***E.cruoralis***5***E.xanthocrossa*. **6***Loxostegesticticalis***7***L.deliblatica***8***Achyramassalis***9***Sitochroaumbrosalis***10***S.palealis*. Scale bar: 0.5 mm.

Species of *Emphylica* have a conical frons (Figs 2–5), similar to species of *Achyra*, *Loxostege* and *Sitochroa* (Figs 6–10), and by this differing from most genera of Pyraustinae. They can be best distinguished from the genera mentioned above in male genitalia by the narrowly triangular to trapezoid, sparsely to moderately setose uncus, the scale-liked editum, the sclerotized ventral process of the sella pointing towards the ventral margin of the valva, the nearly U-shaped juxta, the well-developed, distally rounded saccus, and the interlaced spicules in the phallus. In female genitalia, the antrum is sclerotized and the signum is rhombic.

#### Description.

**Figure 11.**
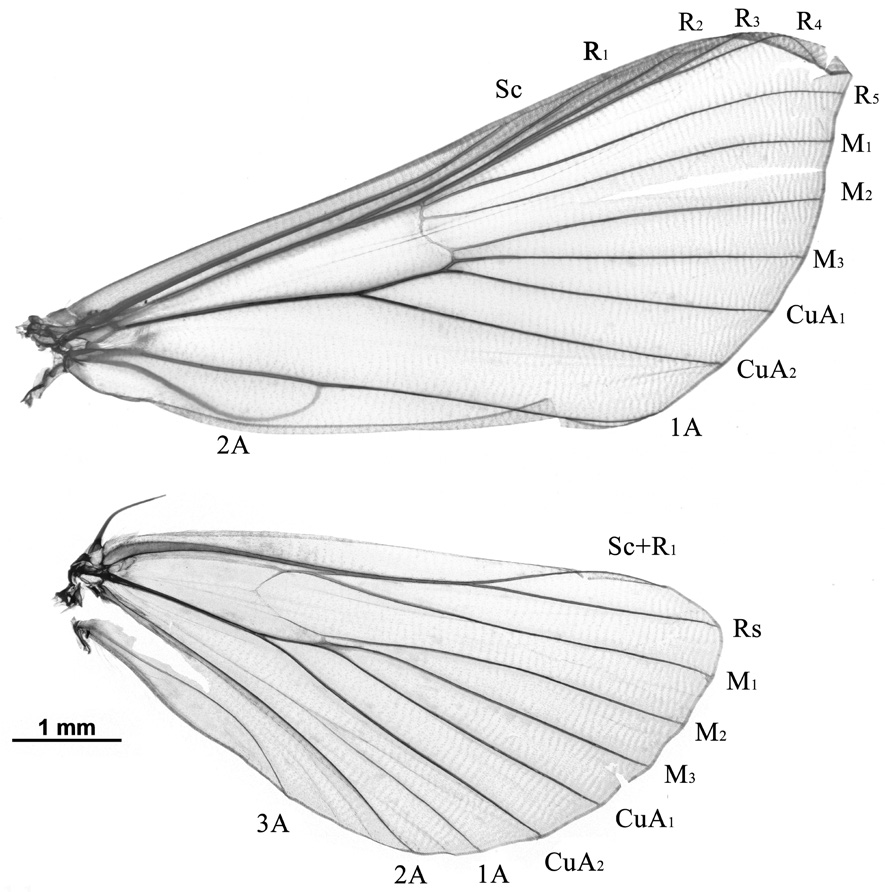
Wing venation of *Emphylicacrassihamata*.

**Head.** Frons conical. Vertex with moderately raised scales projecting between antennae. Labial palpus slightly upwardly curved, approximately twice as long as diameter of eye; first segment with white scales at base; second segment obliquely directed upward; third segment long, porrect. Maxillary palpus prominent, curved upward. Proboscis well developed, with creamy white scales at base. Antenna in male with cilia shorter than or as long as width of corresponding flagellomeres. **Thorax.** Dorsal side whitish brown to brown; ventral side whitish to pale yellow. Legs unmodified, hindleg with basal inner spur longer than apical inner spur, approximately three times as long as basal outer spur. **Wings.** Forewing elongate-triangular, costa straight to near apex, then slightly arched to apex; apex sharp; termen weakly arched, oblique to tornus; dorsum straight; upperside usually with reddish or pale brown scales; frenulum hook in male well developed, retinaculum made up a tuft of curved bristles from below base of discal cell. Hindwing broad, fan-shaped; terminal margin usually brown; frenulum simple in male, with 2 acanthae in female. Wing venation as in Fig. 11. **Abdomen.** Apical margin of segments tinged with yellowish white. **Male genitalia.** Uncus narrowly triangular to narrowly trapezoid, more or less bulging near base. Tegumen trapezoid. Vinculum U-shaped. Saccus well developed, rounded triangular, approximately as long as uncus. Valva of medium width, tongue-shaped, slightly narrowed or tapering to rounded apex, ventral margin straight to slightly curved; transtilla short, triangular, usually with sclerotized ventral process extending to distal end of juxta; costal sclerotized band broad, slightly curved; dorsal sella short, lamellar, set with thick scale-like setae forming editum, more or less curved, apically with several filaments; ventral sella strongly sclerotized, usually perpendicularly pointing towards ventral margin of valva, usually curved apically; sacculus broad, usually with pointed sclerotized dorsal process (absent in *E.xanthocrossa*). Juxta usually U-shaped, distal arms sclerotized. Phallus tubular, usually with interlaced cornuti, in distal end with spine-like or teeth-like area of teeth. **Female genitalia.** Ovipositor lobes flat, densely setose. Posterior apophysis simple, anterior apophysis usually bulging near basal third. Antrum sclerotized. Ductus seminalis originating from anterior end of colliculum. Ductus bursae long and slender, more than 1.5× as long as diameter of corpus bursae. Corpus bursae globular, spinulose; accessory bursa present, arising from corpus bursae mediolaterally; signum narrowly rhombic to sea-star-shaped.

#### Biology.

All of the Chinese material has been collected during the night at light. Host information is currently unavailable.

#### Distribution

(Fig. 24). India, China, Australia.

### Key to species of *Emphylica* based on males

**Table:** 

1	Wingspan usually less than 15 mm (Fig. 15). Uncus concave distally, ductus ejaculatorius originating from the middle of the phallus (Fig. 19A, C)	***E.xanthocrossa* Turner, 1913**
–	Wingspan usually larger than 17 mm. Uncus not concave distally, ductus ejaculatorius originating from the anterior end of the phallus	**2**
2	Ground colour of the wings whitish (Fig. 12). Uncus triangular, ventral sella reaching ventral margin of valva or beyond (Fig. 16A)	***E.diaphana* (Caradja & Meyrick, 1934), comb. n.**
–	Ground colour of the wings reddish brown or yellow. Uncus trapezoid, ventral sella not reaching ventral margin of valva (Figs 17A, 18A)	**3**
3	Basal 2/3 of forewing predominantly reddish brown (Fig. 14). Ventral sella triangular, overlaid by a folded, distally blunt process (Fig. 18A, B)	***E.cruoralis* (Warren, 1895), comb. n.**
–	Basal 2/3 of forewing yellow, sprinkled with reddish brown scales (Fig. 13). Ventral sella hook-like (Fig. 17A, B)	***E.crassihamata* sp. n.**

### Emphylica diaphana

Taxon classificationAnimaliaLepidopteraCrambidae

(Caradja & Meyrick, 1934) comb. n.

**Figures 12–15.**
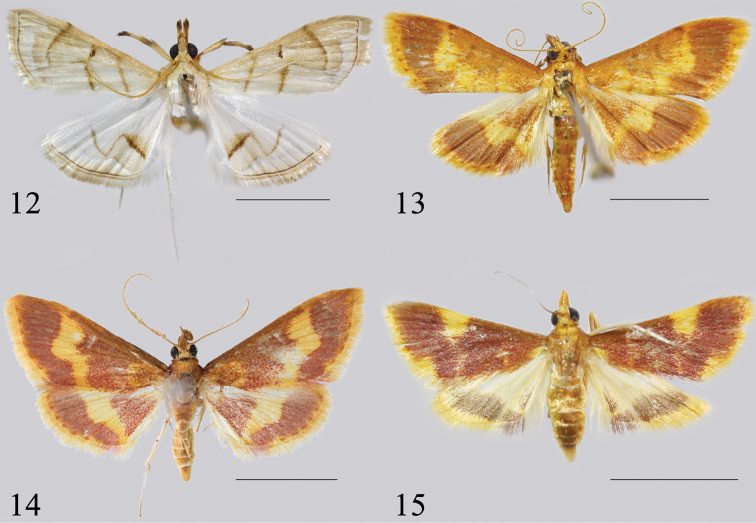
Adults of *Emphylica* spp. **12***E.diaphana*, male (Hexi, Fujian) **13***E.crassihamata*, holotype, male (Shixing, Guangdong) **14***E.cruoralis*, male (Khasis, India) **15***E.xanthocrossa*, female (Hidden Valley, Western Australia). Scale bar: 5.0 mm.

**Figures 16–17.**
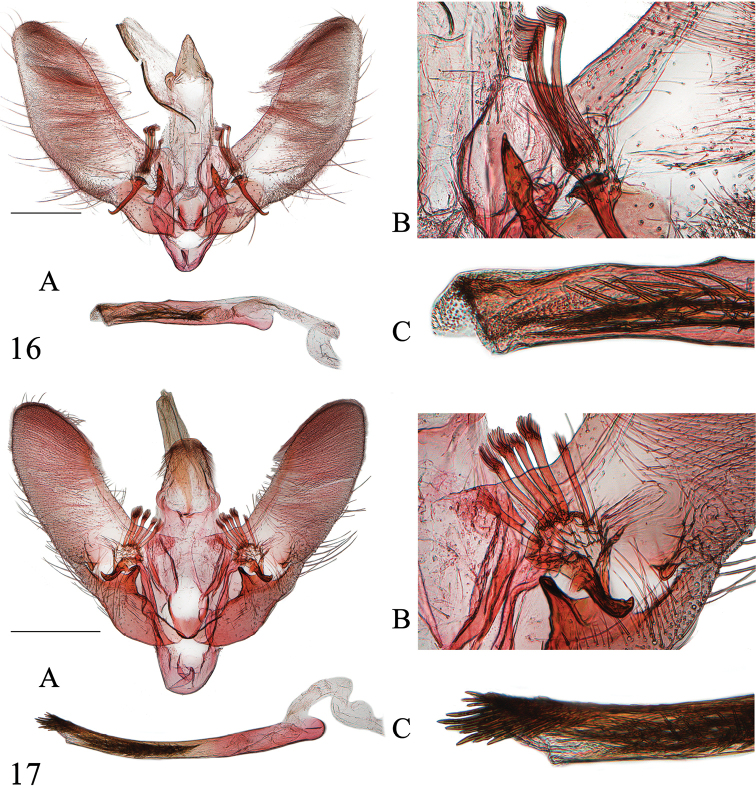
Male genitalia of *Emphylica* spp. **16***E.diaphana*, Chongqing (genitalia slide no. SYSU0965) **17***E.crassihamata*, Guangdong (genitalia slide no. SYSU0933) **A** Whole genitalia **B** Base of valva dorsally **C** Distal part of phallus. Scale bar: 0.5 mm.

Figs 2 , 12 , 16 , 20 , 24

Loxostege diaphana Caradja & Meyrick, 1934: 164.

#### Material examined.

**CHINA, Fujian**: 1♂, Letu rain forest, Hexi, Nanjing, 24.90N, 117.22E, alt. 270 m, 10.VII.2014, leg. Zhang Dandan, genitalia slide no. SYSU1040, molecular voucher no. SYSU-LEP0263; **Guangdong**: 1♂, Sanyue Reserve, 24.03N, 111.57E, alt. 272 m, 6.VII.2013, leg. Chen Xiaohua, genitalia slide no. SYSU1041, molecular voucher no. SYSU-LEP0264; **Hainan**: 1♂, Mt. Limushan, 5.V.2011, leg. Zhang Dandan and Yang Lijun; **Chongqing**: 1♀, Daheba, Mt. Jinfoshan, alt. 800–850 m, 15.VII.2010, leg. Du Xicui and Song Lifang, genitalia slides no. SYSU0969; 1♂, Daheba, Mt. Jinfoshan, alt. 800–850 m, 16.VII.2010, leg. Du Xicui and Song Lifang, genitalia slides no. SYSU0965.

#### Diagnosis.

*Emphylicadiaphana* resembles other *Emphylica* species in the conical frons and the scale-like setae of the sella. It can be best distinguished from its congeners by the whitish ground colour suffused with pale brown scales on both wings, the dark brown lines at termen, in male genitalia by the triangular, distally narrowly-rounded uncus bearing only few short setae and the long, strongly sclerotized ventral sella usually projecting beyond the ventral margin of the valva. In female genitalia of *E.diaphana*, the antrum is strongly sclerotized, shorter than the length of the anterior apophysis, slightly wider than the ductus bursae; the maximal length of the signum is approximately 2/3 as long as the diameter of the corpus bursae; the two opposing angles of the signum without carinae are well-developed, almost as long as the other two. In *E.xanthocrossa* the antrum is broad, lightly sclerotized, no more than twice as wide as the ductus bursae; the two opposing angles of the signum without carinae are fairly short; in *E.crassihamata* the antrum is longer than the anterior apophysis, in *E.cruoralis* the antrum is as long as the anterior apophysis and the signum of both species is small (less than half of the diameter of the corpus bursae).

#### Redescription

(Figs 2, 12). **Head.** Frons and vertex pale yellow mixed with few white scales. Antenna pale brown, cilia in male less than half as wide as corresponding flagellomeres. Labial palpus brown mixed with pale yellow medially, with white scales at base. Maxillary palpus brown, pale brown at tip. **Thorax.** Whitish brown at dorsum, whitish ventrally. Foreleg: femur yellow, ventrally white, tibia pale brown, first tarsus pale brown, second tarsus white mixed with pale brown, third and fourth tarsus dark brown, fifth tarsus white. Midleg: femur dorsally and tibia pale yellow, remainder whitish; inner spur about twice as long as outer one. Hindleg: pale yellow; basal inner spur approximately three times as long as basal outer spur; apical inner spur about twice as long as apical outer spur. Wingspan 17–19 mm. Forewing whitish sprinkled with pale brown. Antemedial line pale brown from basal third of costa, oblique, reaching beyond basal 1/4 of dorsum; reniform stigma a short streak, pale brown mixed with dark brown scales posteriorly; postmedial line brown, arched from beyond basal 2/3 of costa to about 2/3 of CuA_1_, bent inwards to posterior angle of cell, then oblique to beyond half of dorsum; subterminal band whitish, with anterior 1/3 faint, gradually narrowed to tornus; termen line dark brown; fringe white at base and brown posteriorly. Hindwing white; postmedial line brown, darkened and thickened posteriorly, from base of Rs, weakly curved to about 2/3 of CuA_1_, bent inwards to beyond base of CuA_1_, then bent at right angle outwards to 3/4 of inner margin, latter section with pale brown anteriorly; subterminal line faint, from about 7/10 of M_2_, weakly curved inwards to 3/4 of CuA_2_, then slightly darkened, curved outwards to end of 2A; area between posterior part of posterior line and subterminal line sprinkled with few pale brown scales; terminal margin with few pale brown scales medially; termen edged by dark brown line; fringe as in forewing, entirely white near tornus. **Abdomen.** Dorsal segments pale brown, apical margins of basal four segments brown, edged by white scales, apical margins of remainder segments with white scales, 8th segment with two small dark brown spots posterolaterally; segments whitish ventrally. **Male genitalia** (Fig. 16). Uncus narrow, triangular, bearing few hair-like setae at distal third. Valva with ventral margin curved, gradually narrowed towards obtusely rounded apex; costal sclerotized band wide, slightly curved to 2/3 of dorsal margin; sacculus broad, distal half expanded, with a pointed, strongly sclerotized process projecting dorsally, sparsely setose; dorsal sella sub-rectangular; ventral sella long and slender, strongly sclerotized, distally curved, usually reaching or extending beyond ventral margin of valva. Juxta plate-shaped with lateral part strongly sclerotized, distal half slightly divided medially. Phallus tubular, straight, approximately 4/5 as long as length of valva, distal half with interlaced spicules, distal end dorsally with several small, teeth-like spines. **Female genitalia** (Fig. 20). Anterior apophysis with triangular expansion near basal third. Antrum cup-shaped, slightly wider than ductus bursae, medially somewhat constricted. Ductus bursae slender, approximately 2× as long as diameter of corpus bursae; colliculum well developed, slightly sinuate laterally, approximately as long as antrum. Corpus bursae globular; accessory bursa globular; signum sea-star-shaped, with two angles bearing carinae disconnected medially, distally pointed, other two angles well developed, distally rounded.

#### Distribution

(Fig. 24). China (Fujian, Guangdong, Hainan, Chongqing).

#### Remarks.

This species was formerly placed in the genus *Loxostege*, probably based on the conical frons. However, genitalia traits of *Loxostege* moths, e.g. the cylindrical uncus with dense, scale-like setae, the few hair-like setae of the dorsal sella, the ventrobasally directed ventral sella and the usually coiled ductus bursae, are different in *Emphylicadiaphana*. Although in appearance the wing colour and pattern of this species are somewhat dissimilar to those of other *Emphylica* species, the genitalia traits agree with the diagnostic characters of *Emphylicaxanthocrossa* Turner, the type species. Moreover, according to the molecular phylogeny, this species was inferred as terminal lineage within *Emphylica*, rather than in *Loxostege*. Consequently, this species is considered as correctly placed in *Emphylica*.

### Emphylica crassihamata

Taxon classificationAnimaliaLepidopteraCrambidae

Chen & Zhang sp. n.

http://zoobank.org/F348D37E-4CA9-41A5-89CE-09866C0B0B4F

Figs 3 , 11 , 13 , 17 , 21 , 24

#### Material examined.

**Type material.** Holotype ♂ (Fig. 13); **CHINA, Guangdong**: Chebaling National Nature Reserve, Shixing County, 24.72N, 114.26E, alt. 496 m, 28.V.2017, leg. Chen Kai. Paratypes: **Hunan**: 1♂, Mt. Huilongshan, Zixing, 26.08N, 113.39E, alt. 886 m, 8.VI.2016, leg. Chen Kai and Duan Yongjiang; 1♂, Jinyinpu, Bamianshan Reserve, Guidong County, 25.97N, 113.71E, alt. 973 m, 16.VI.2015, leg. Chen Kai; 2♀, Gaowangjie National Nature Reserve, Guzhang County, 28.66N, 110.08E, alt. 890 m, 18.VI.2017, leg. Zhang Dandan, genitalia slides no. SYSU0994 (molecular voucher no. SYSU-LEP0191), 0957; **Guangdong**: 1♂, same data as holotype; 2♂, idem except leg. Duan Yongjiang, genitalia slide no. SYSU0993, molecular voucher no. SYSU-LEP0190; 2♂, idem except leg. Kou Zongqing, genitalia slide no. SYSU0933.

#### Diagnosis.

In appearance, *E.crassihamata* resembles *E.cruoralis* in the reddish brown subterminal band, but can still be recognized by the predominantly yellow basal 2/3 of the forewing sprinkled with reddish brown scales and the presence of a faint antemedial line on the forewing and postmedial lines on both wings. In male genitalia it differs from *E.diaphana* and *E.xanthocrossa* by the distally rounded, moderately setose uncus, the pointed and recurved dorsal process of the sacculus, and the long and slender phallus, which is longer than the length of the valva; from *E.cruoralis* it differs by the wider distal uncus, the small triangular, strongly sclerotized process near the distal sacculus as well as the hook-like ventral sella. In female genitalia, the sclerotized antrum is approximately 1.5× as long as the anterior apophysis whereas in *E.cruoralis* the sclerotized antrum is as long as the anterior apophysis.

#### Description

(Figs 3, 13). **Head.** Frons and vertex yellowish brown, frons with cream white stripe laterally. Antenna yellowish brown, cilia in male as long as width of corresponding flagellomeres. Labial palpus brown with white scales at base. Maxillary palpus brown. **Thorax.** Saffron dorsally, pale yellow ventrally. Foreleg: femur brown; tibia brown and white alternately; tarsi white except distal three brown. Midleg: femur pale brown; tibia yellow dorsally, white ventrally, outer spur half as long as inner one; tarsi white ventrally, pale yellow dorsally. Hindleg: femur pale brown; tibia yellow, basal inner spur in male about three times as long as basal outer spur, apical inner spur about twice as long as apical outer spur; tarsi pale yellow. Wingspan 17.5–18.5 mm. Forewing yellow edged by reddish brown subterminal band, sprinkled with reddish brown scales from base to postmedial line, slightly darkened along costal margin, veins covered with reddish brown scales terminally, terminal band narrow, saffron; antemedial line reddish brown, curved outwards from basal fourth of costa to about basal third of dorsum; orbicular stigma faint, dark brown; reniform stigma straight, strip-like, dark brown; postmedial line reddish brown, weakly sinuate from 3/4 of costa to base of M_2_, bent inwards to base of CuA_2_, then curved outwards to about middle of dorsum; inner margin of subterminal band nearly parallel to postmedial line; underside with ground colour as on upperside but paler; fringe saffron mixed with pale yellow scales, mostly reddish brown at tornus. Hindwing with costal margin translucent white to 2/3 of costa, basal half medially pale reddish brown, followed by pale yellow band, outer margin sinuate, edged by reddish brown subterminal band, terminal band narrow, saffron, veins with reddish brown scales terminally; postmedial line indistinct; fringe as in forewing; underside paler than upperside especially in basal half. **Abdomen.** Brown dorsally, whitish ventrally, apical margins of segments tinged with white.

#### Male genitalia

(Fig. 17). Uncus bulging at base, gradually narrowed towards obtusely rounded apex, maximal width approximately 2× minimal width, bearing hair-like setae on distal half. Valva evenly wide medially, slightly tapering towards apex, with a small triangular sclerotized process beyond distal end of sacculus; transtilla triangular; costal sclerotized band wide, slightly expanded to 2/3 of dorsal margin; sacculus broad, distal third expanded and bearing a strongly sclerotized, hook-like process; dorsal sella quadrate; ventral sella hook-like, strongly sclerotized. Juxta U-shaped with two slender arms, thickened and fused in basal half. Phallus long and slender, slightly curved upward, approximately 1.25× length of valva, distal half with interlaced spicules on vesica, distal end with several pointed cornuti dorsally. **Female genitalia** (Fig. 21). Anterior apophysis slightly bulging near basal third. Antrum long, funnel-shaped, thickened and strongly sclerotized distolaterally, approximately 1.5× as long as anterior apophysis. Ductus bursae slender, as wide as anterior part of antrum, approximately 1.8× as long as diameter of corpus bursae; colliculum slightly narrowed posteriorly. Corpus bursae globular, spinulose; accessory bursa arising from middle side of corpus bursae; rhombic signum small, maximal length approximately 1/3 as long as diameter of corpus bursae, with two opposing angles bearing carinae disconnected medially, other two angles triangular, distally blunt.

#### Etymology.

The specific name is derived from the Latin *crassi*- = thick and *hamata* = hook-like, referring to the thick, hook-like ventral sella.

#### Distribution

(Fig. 24). China (Hunan, Guangdong).

### Emphylica cruoralis

Taxon classificationAnimaliaLepidopteraCrambidae

(Warren, 1895) comb. n.

**Figures 18–19.**
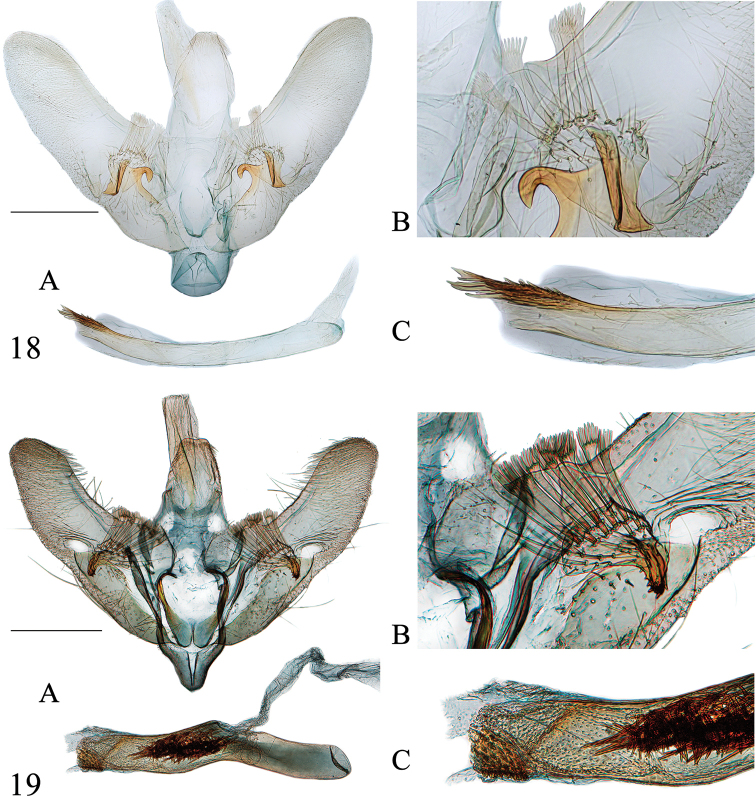
Male genitalia of *Emphylica* spp. **18***E.cruoralis*, Khasis (PyralidaeNHMUK Slide no. 10935) **19***E.xanthocrossa*, Queensland (genitalia slide no. ANIC21185) **A** Whole genitalia **B** Base of valva dorsally **C** Distal part of phallus. Scale bar: 0.5 mm.

Figs 4 , 14 , 18 , 22 , 24

Syllythria cruoralis Warren, 1895: 471.
Pyrausta cruoralis (Warren, 1895): Hampson 1896: 432.

#### Material examined.

**Type material.** Lectotype (here designated) ♂: **INDIA, Meghalaya**: Khasis, Mar.1894, Nat. Coll., PyralidaeNHMUK Slide no. 10935 (NHMUK).

#### Other material examined.

**INDIA, Meghalaya**: 7♂ (Fig. 14), Khasia Hills, Assam, Nissary (NHMUK); 8♂, Assam, Khasis, Nat. Coll. (NHMUK); 1♂, same data as type (NHMUK); 1♂, Khasis Hills, Assam (NHMUK); 1♂, Khasis Hills (NHMUK); **CHINA, Tibet**: 1♀, air-raid shelter, Beibeng Village, Medog County, 29.24N, 95.17E, alt. 750 m, 31.VII.2018, leg. Qi Mujie, genitalia slide no. ZDD12100 (molecular voucher no. SYSU-LEP0377) (NKU).

#### Diagnosis.

*Emphylicacruoralis* resembles *E.crassihamata* in the reddish brown subterminal band and the saffron fringe. The differences between the two species are provided in the diagnosis of *E.crassihamata*. In appearance, *E.cruoralis* can be best recognized within the genus by the yellow postmedial band of the forewing, in male genitalia by the narrow trapezoid uncus with hair-like setae at distal third, the large, thumb-shaped, weakly sclerotized process of the ventral valva near the distal sacculus as well as the triangular ventral sella overlaid by a folded, distally blunt process. In female genitalia, it resembles *E.crassihamata* except for the less sinuate distolateral antrum and the relatively shorter and wider antrum anteriorly.

#### Redescription

(Figs 4, 14). **Head.** Frons and vertex yellowish brown. Antenna pale yellowish brown, cilia in male as long as width of corresponding flagellomeres. Labial palpus yellowish brown with white scales at base. Maxillary palpus yellowish brown. **Thorax.** Yellowish brown dorsally, whitish ventrally. Foreleg: femur brown; tibia brown and white alternately; tarsi white except for pale brown distal three. Midleg: femur pale brown; tibia yellow on dorsum, white ventrally, outer spur half as long as inner one; tarsi white ventrally, pale yellow dorsally. Hindleg: yellowish brown, basal inner spur in male about three times as long as basal outer spur, apical inner spur about twice as long as apical outer spur. Wingspan 16–19 mm. Forewing with reddish brown ground colour, except for saffron circle at base and sinuate, saffron postmedial band, narrowing towards costa and dorsum; costa straight, slightly arched to apex, brown at basal 2/3, yellowish brown at distal third; terminal margin with narrow, saffron intermittent band; fringe saffron, mixed with reddish brown near tornus. Hindwing with costal margin translucent white to 2/3 of costa, basal half medially reddish brown, followed by yellow postmedial band, narrowing towards tornus, outer margin sinuate, edged by reddish brown subterminal band, terminal band narrow, intermittent, saffron, posterior margin pale yellow; fringe as in forewing. **Abdomen.** Dorsally yellowish brown, apical margins of segments tinged with white. **Male genitalia** (Fig. 18). Uncus slightly bulging at base, gradually narrowed towards truncate apex, maximal width approximately 3× minimal width, distal third with hair-like setae. Valva evenly wide medially, slightly tapering towards rounded apex, ventral margin with weakly sclerotized, thumb-shaped process projecting basally near distal end of sacculus; costal sclerotized band moderately wide, slightly curved to beyond 2/3 of dorsal margin; sacculus broad, distal third expanded and bearing a strongly sclerotized, hook-like process; ventral sella triangular, weakly sclerotized, overlaid by strongly sclerotized, folded, distally blunt process. Juxta U-shaped with two narrow tapering arms, basally broadened. Phallus as in *E.crassihamata* (without interlaced spicules of vesica in Fig. 18). **Female genitalia** (Fig. 22). As in *E.crassihamata* except: distolateral antrum less sinuate, relatively shorter and wider anteriorly; ductus bursae narrower than width of anterior part of antrum.

#### Distribution

(Fig. 24). India (Meghalaya), China (Tibet).

#### Remarks.

This species was formerly placed in the genus *Pyrausta*. However, both the molecular phylogeny and the genital traits suggested that it should be placed in *Emphylica*. According to the male genitalia (Fig. 18) of the type specimen of *Emphylicacruoralis* (Warren, 1895), **comb. n.**, this species agrees with diagnostic characters of *Emphylica*. It differs from *Pyrausta* by the conical frons, in male genitalia by the the presence of an editum made of modified, scale-like setae, the more anteriorly positioned and ventrally directed and sclerotized sella, and the more strongly developed sclerotized dorsal process of the sacculus, and in female genitalia (Fig. 22) by the strongly sclerotized antrum.

### Emphylica xanthocrossa

Taxon classificationAnimaliaLepidopteraCrambidae

Turner, 1913

Figs 5 , 15 , 19 , 23 , 24

Emphylica xanthocrossa Turner, 1913: 159.

#### Material examined.

**Type material.** Holotype, ♀: **AUSTRALIA, Northern Territory**: P[ort]. Darwin, Nov.[19]08, leg. F.P. Dodd, genitalia slide no. P232 (ANIC).

#### Other material examined

(ANIC). **AUSTRALIA, Northern Territory**: 1♂, 16.19S, 136.05E, 36 km SW of Borroloola, NT, 4.Nov.1975, leg. E.D. Edwards, K. Maes Gen. Prep. nr.: 20741; genitalia slide no. ANIC18161; 1♂, 16.10S, 136.15E, Goose Lagoon, 11 km SW by S Borroloola, NT, 31.Oct.1975, E.D. Edwards leg.; 1♂, Humpty Doo, N.T., Light Trap, 10.Nov.1959, E.B. Boerema leg.; 2♂, 16.40S, 135.51E, Bessie Spring, 8 km ESE of Cape Crawford, NT, 26.Otc.1975, E.D. Edwards leg., genitalia slide no. P707; 1♀, 16.41S, 135.44E, Cape Crawford road junction, NT, 29.Mar.1995, E.D. Edwards and M. Matthews leg.; **Queensland**: 1♂, 15.45S, 144.15E, 2 km NNW of Jowabinna, 17.I.1994, E.D. Edwards and P. Zborowski leg., genitalia slide no. ANIC21185; 1♀, 12.42S, 142.30E, Moonlight creek, QLD, 13.Nov.1993, at light, P. Zborowski and M. Horak leg., K. Maes Gen. Prep. nr.: 20742, genitalia slide no. ANIC18162; 1♂, 12.40S, 142.40E, Batavia Downs, QLD, 22-23.Nov.1992, at light, P. Zborowski and A. Calder leg.; 1♂, 12.40S, 142.41E, Batavia Downs, QLD, 11.Dec.1992, at light, P. Zborowski and W. Dressler leg.; **Western Australia**: 1♀, 15.77S, 128.75E, Hidden Valley, Kununurra, III.2016, P.M. Heath leg., genitalia slide no. ANIC21184, molecular voucher no. SYSU-LEP0307; 1♂, Kunnunurra, W.A., 9.Apr.1962, I.F.B. Common leg.; 1♂, Wyndham, W.A., ?.?.[19]30, T.G. Campbell leg.; 1♂, 16.10S, 128.23E, nr Dunham River crossing, WA, 6.Apr.1995, E.D. Edwards and M. Matthews leg.

**Figures 20–23.**
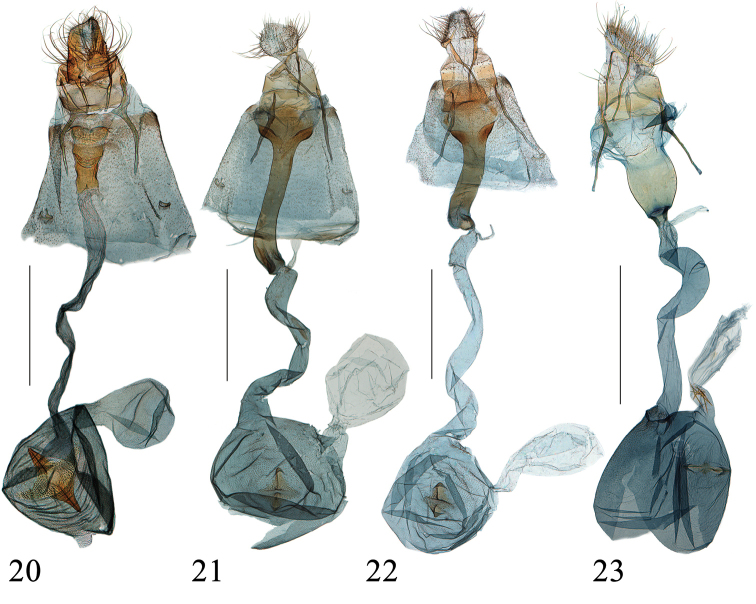
Female genitalia of *Emphylica* spp., ventral views **20***E.diaphana*, Chongqing (genitalia slide no. SYSU0969) **21***E.crassihamata*, Hunan (genitalia slide no. SYSU0957) **22***E.cruoralis*, Tibet (genitalia slide no. ZDD12100) **23***E.xanthocrossa*, Queensland (genitalia slide no. ANIC18162). Scale bar: 1.0 mm.

#### Diagnosis.

*Emphylicaxanthocrossa* resembles *E.crassihamata* and *E.cruoralis* in the saffron fringe, the conical frons and the U-shaped juxta. It can be best distinguished from its congeners by the smaller wingspan (less than 15 mm), the triangular saffron spot on the forewing costa postmedially, the smoky brown subterminal margin of the hindwing, in male genitalia by the distally concave uncus, the spinulose ventral sella, the absence of a dorsal process on the sacculus, the larger juxta, the broad and slightly sinuate phallus and the ductus ejaculatorius originating from the middle of the phallus. In female genitalia, the antrum is moderately sclerotized, bottle-shaped, the two opposing angles of the signum without carinae are short, whereas in *E.diaphana*, *E.crassihamata* and *E.cruoralis* the antrum is strongly sclerotized and the two opposing angles of the signum without carinae are almost as long as the other two.

#### Redescription

(Figs 5, 15). **Head.** Frons and vertex pale yellowish brown, frons with cream white stripe laterally. Antenna brown, cilia in male less than half width of corresponding flagellomeres. Labial palpus brown and pale yellow alternately with white scales at base, pale yellow at tip. Maxillary palpus yellowish brown. **Thorax.** Pale yellow dorsally, whitish ventrally. Foreleg: yellow except distally white tibia and alternately yellow and white tarsi. Midleg: pale yellow, tibia and tarsi white ventrally; inner spur about twice as long as the outer one. Hindleg: yellowish white; basal outer spur reduced; apical inner spur about 3× as long as apical outer spur. Wingspan 13–14 mm. Forewing reddish brown, with a large triangular to sub-quadrate saffron spot on costa postmedially, a small saffron spot at base of dorsum and a semi-oval saffron patch at termen near tornus; antemedial and postmedial lines almost invisible except near dorsum; costal margin pale brown except at yellow spot; terminal margin mixed with saffron; fringe saffron; underside as upper side but paler, translucent at dorsum. Hindwing with costal margin translucent white to 2/3 of costa; termen arched to 1/2 then strongly oblique to tornus; distal third smoky brown except for saffron terminal area from apex to 1A; below posterior angle of cell covered with few brown scales, and a triangular patch of brown scales near tornus; remainder pale yellow; fringe as in forewing except brown near tornus; underside pale yellow. **Abdomen.** Dorsally covered with saffron scales, whitish ventrally, apical margin of segments tinged with yellowish white. **Male genitalia** (Fig. 19). Uncus with lateral margin slightly bulging at base, then gradually narrowed to concave apex, setose on distal third. Valva evenly wide in middle, tapering to rounded apex; transtilla triangular; costal sclerotized band wide, slightly curved to 2/3 of dorsal margin; sacculus broad, distal half moderately expanded; dorsal sella sub-rectangular; ventral sella triangular, slightly flexed and curved, spinulose, distally blunt. Juxta large U-shaped with two strongly sclerotized, curved, tapering distal arms, thickened basally and medially divided. Phallus tubular, slightly sinuate, approximately 1.1× length of valva, distal fourth spinulose, apically with dense, teeth-like spines ventrally; ductus ejaculatorius originating from middle of phallus; vesica with bundle of interlaced spicules. **Female genitalia** (Fig. 23). Posterior apophysis long and slender, approximately 4/5 as long as anterior apophysis. Anterior apophysis with triangular expansion near basal third. Antrum moderately sclerotized, bottle-shaped, slightly bulging medially. Ductus bursae slender, approximately 1.4× as long as diameter of corpus bursae; colliculum slightly narrowed medially. Corpus bursae globular; rhombic signum small, maximal length approximately 1/3 as long as diameter of corpus bursae, with two distally pointed opposing angles bearing carina disconnected medially, other two angles small, indistinct.

#### Distribution

**Figure 24.**
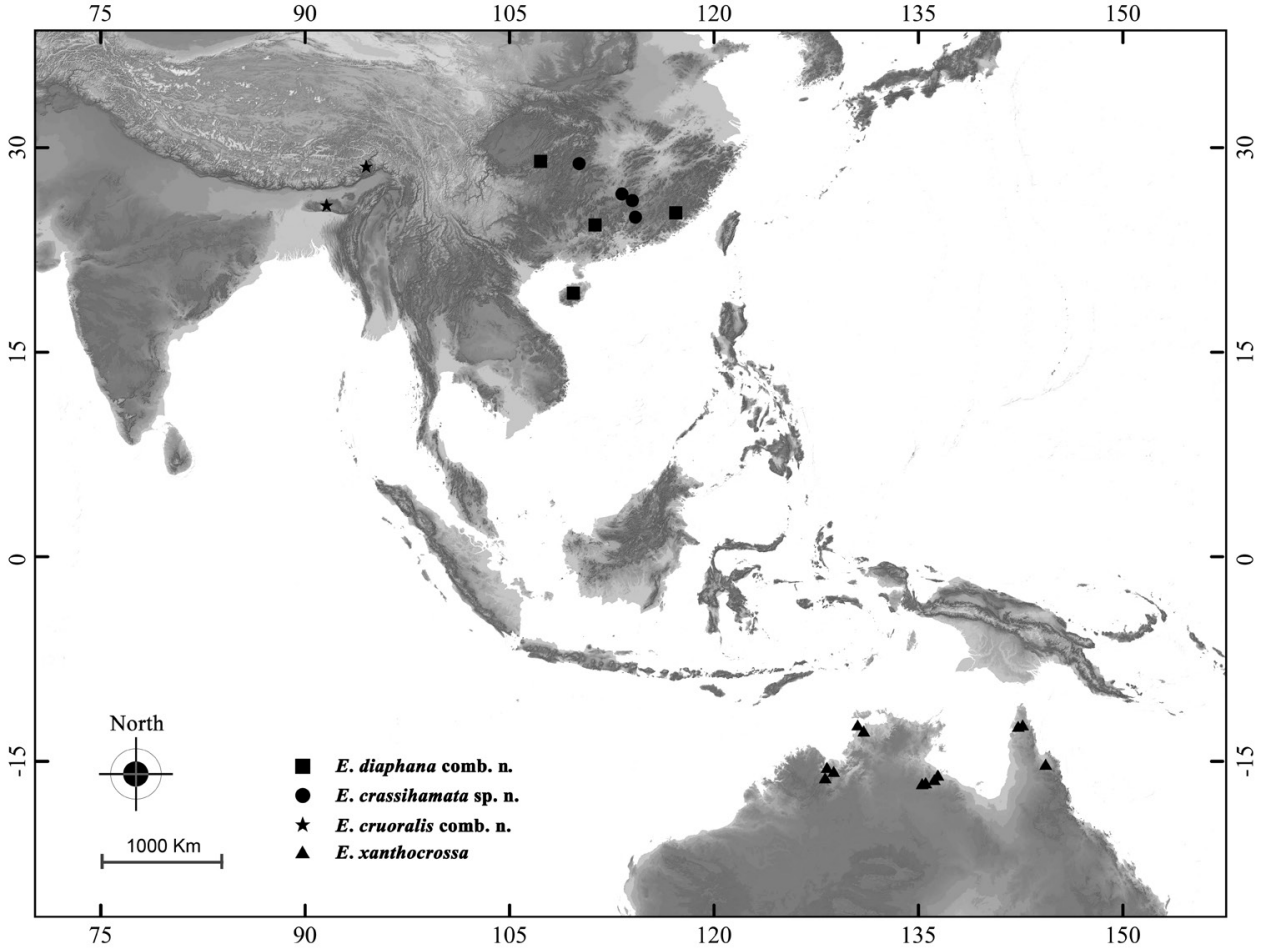
Distribution map of *Emphylica* spp.

(Fig. 24). Australia (Northern Territory, Queensland, Western Australia).

## Discussion

Based on the molecular phylogeny, the clade *Achyra* + (*Loxostege* + *Sitochroa*) is a sister group to *Emphylica*. All these four genera have a similar conical frons. Species of *Achyra* can be distinguished from *Emphylica* in male genitalia by the narrow triangular uncus laterally set with dense hair-like setae, the dense simple setae of the sella and in female genitalia by the presence of a second signum and the broad base of the ductus seminalis. Species of *Loxostege* differ from *Emphylica* in male genitalia by the cylindrical uncus set with scale-like setae, the few simple setae of the sella and in female genitalia by the long and coiled ductus bursae with a sclerotized base. Species of *Sitochroa* are best distinguished from *Emphylica* in male genitalia by the ventral sella with two hook-like, basally curved processes and the strongly sclerotized process extending from the phallus apically, and in female genitalia by the twisted sclerite near the posterior end of the ductus bursae. *Hahncappsia*, *Neohelvibotys* and *Helvibotys*, mainly distributed in the Nearctic and Neotropical regions, are not included in the current phylogenetic analysis. External characters of these three genera are similar to those of *Achyra*, *Loxostege* and *Sitochroa* (Munroe, 1976a). The morphological details were provided by Munroe (1976a, b). In male genitalia, *Emphylica* moths can be best distinguished from species of *Hahncappsia*, *Neohelvibotys* and *Helvibotys* by the usually trapezoid uncus, the thick, scale-like editum and the well-developed, distally rounded saccus. Some species of *Hahncappsia* have a row of scale-like setae on the sella and the sclerotized process of the sacculus is similar to those of *Emphylica*. The relationships between all these genera need to be further studied.

The monophyly of *Emphylica* is robustly supported by the results of the molecular analysis. Four species can be recognized as members of *Emphylica* based on the series of morphological characters provided above in the diagnosis of the genus. According to the tree topology (Fig. 1), *E.crassihamata* + *E.cruoralis* and *E.xanthocrossa* form a separate group, but with relatively low support in the ML analysis (BS = 40). Both BI and ML analyses show the same topologies, suggesting that *E.crassihamata* and *E.cruoralis* are more closely related to *E.xanthocrossa* than to *E.diaphana* which makes good sense with respect to the wing colouration. Within the genus, *E.crassihamata* is most closely related to *E.cruoralis* in the hindwing pattern (Figs 13, 14) and the genitalia (Figs 17, 18, Figs 21, 22).

*Emphylica* is recorded for the first time from outside Australia. The current study shows that *Emphylica* species occur in Southern China, the northwest of India and the north of Australia. There is as yet no record of *Emphylica* species in Indochina and the Malay Archipelago. Noteworthily, there are three *Pyrausta* spp., probably congeneric with *Emphylica*, recorded in Borneo (see http://www.pyralidsofborneo.org/index.php?cruoralis; http://www.pyralidsofborneo.org/index.php?sp4-17; http://www.pyralidsofborneo.org/index.php?sp5-10). The specimen identified as *P.cruoralis* is very similar to *E.cruoralis* in wing pattern, but with much narrower yellow bands on both wings. The wing pattern of another specimen identified as *P.* sp4 is almost the same as the former specimen, but the wingspan is similar to that of *E.xanthocrossa*. The last specimen identified as *P.* sp5 also has yellow bands of both wings similar to those of *E.cruoralis*. Unfortunately, the frons of these specimens cannot be observed, and no image of genitalia or genetic data can be accessed. Considering the similarity in the wing pattern, it would not be a surprise if these three taxa turn out to be congeneric with *Emphylica* species.

## Supplementary Material

XML Treatment for Emphylica

XML Treatment for Emphylica diaphana

XML Treatment for Emphylica crassihamata

XML Treatment for Emphylica cruoralis

XML Treatment for Emphylica xanthocrossa
